# Pannexin 2 protein expression is not restricted to the CNS

**DOI:** 10.3389/fncel.2014.00392

**Published:** 2014-11-25

**Authors:** Maxence Le Vasseur, Jonathan Lelowski, John F. Bechberger, Wun-Chey Sin, Christian C. Naus

**Affiliations:** Department of Cellular and Physiological Sciences, The Life Science Institute, University of British ColumbiaVancouver, BC, Canada

**Keywords:** pannexin 2, gap junction, gene transcription, protein expression, protein distribution, central nervous system (CNS), mouse, mRNA

## Abstract

Pannexins (Panx) are proteins homologous to the invertebrate gap junction proteins called innexins (Inx) and are traditionally described as transmembrane channels connecting the intracellular and extracellular compartments. Three distinct Panx paralogs (Panx1, Panx2 and Panx3) have been identified in vertebrates but previous reports on Panx expression and functionality focused primarily on Panx1 and Panx3 proteins. Several gene expression studies reported that Panx2 transcript is largely restricted to the central nervous system (CNS) hence suggesting that Panx2 might serve an important role in the CNS. However, the lack of suitable antibodies prevented the creation of a comprehensive map of Panx2 protein expression and Panx2 protein localization profile is currently mostly inferred from the distribution of its transcript. In this study, we characterized novel commercial monoclonal antibodies and surveyed Panx2 expression and distribution at the mRNA and protein level by real-time qPCR, Western blotting and immunofluorescence. Panx2 protein levels were readily detected in every tissue examined, even when transcriptional analysis predicted very low Panx2 protein expression. Furthermore, our results indicate that Panx2 transcriptional activity is a poor predictor of Panx2 protein abundance and does not correlate with Panx2 protein levels. Despite showing disproportionately high transcript levels, the CNS expressed less Panx2 protein than any other tissues analyzed. Additionally, we showed that Panx2 protein does not localize at the plasma membrane like other gap junction proteins but remains confined within cytoplasmic compartments. Overall, our results demonstrate that the endogenous expression of Panx2 protein is not restricted to the CNS and is more ubiquitous than initially predicted.

## Introduction

Gap junction proteins are traditionally described as aqueous plasma membrane channels which allow rapid cell-to-cell communication by directly connecting the cytoplasm of adjacent cells. In chordates, connexins (Cxs) are the canonical gap junction proteins while gap junctions in invertebrates are formed exclusively by the evolutionarily unrelated innexin (Inx) family. In 2000, another small gene family named pannexin (Panx) was identified based on sequence homology with the Inx family and was found to be expressed alongside Cxs in chordates (Panchin et al., 2000). Three distinct Panx paralogs (Panx1, Panx2 and Panx3) were initially identified in vertebrates (Panchin et al., 2000; Panchin, 2005; Barbe, 2006) but recent studies showed that Panx1 has been retained as two independent ohnologs in teleost as a result of an ancestral whole genome duplication (Bond et al., 2012; Kurtenbach et al., 2013). Despite the lack of sequence similarity between Inxs/Panxs and Cxs, both families share structural resemblance. Cxs and Panxs both have a predicted topology consisting of four membrane-spanning domains, two extracellular loops, a cytoplasmic loop, and cytoplasmic N- and C-termini (Panchin, 2005). Despite sharing structural resemblance with Cxs, the ability of Panx channels to form gap junctional coupling remains controversial. A few groups reported that Panx1 and Panx3 can form cell-cell junctional channels (Bruzzone et al., 2003; Vanden Abeele et al., 2006; Lai et al., 2007; Ishikawa et al., 2011; Sahu et al., 2014) but their observations were limited primarily to heterologous or over-expression systems and undisputable evidence supporting Panx-based coupling is still lacking. In contrast to Cxs, all three Panxs are glycosylated at their extracellular loops (Penuela et al., 2009) with carbohydrate moieties that sterically hinder the docking of channels from adjacent cells (Boassa et al., 2007). Therefore, it is largely accepted that under physiological conditions, Panx channels primarily form non-junctional membrane channels controlling the exchange of ions and small molecules between the cytoplasm and extracellular space and do not significantly contribute to direct cell-to-cell gap junctional communication (Sosinsky et al., 2011).

Several gene expression profiling studies reported that Panx2 transcriptional activity is largely restricted to the central nervous system in human (Baranova et al., 2004), rat (Bruzzone et al., 2003) and zebrafish (Zoidl et al., 2008; Bond et al., 2012). Minimal Panx2 mRNA levels have also been detected in some non-neural tissues such as the eye, thyroid, prostate, kidney, liver, heart and olfactory epithelium (Bruzzone et al., 2003; Dvoriantchikova et al., 2006; Bond et al., 2012; Zhang et al., 2012) but given the much larger Panx2 mRNA levels found in the CNS, Panx2 transcript and corresponding protein are largely assumed to be primarily expressed in the CNS. In the healthy brain, Panx2 protein was shown to have a complex distribution pattern and is expressed in pyramidal cells and interneurons alike (Zappalà et al., 2007). Interestingly, Panx2 protein was also detected in astrocytes following ischemia in the rat but not in the healthy brain (Zappalà et al., 2007). Panx2 protein is also present in hippocampal neural progenitors and mature neurons both *in vitro* and *in vivo* (Swayne et al., 2010). However, because Panx2 is believed to be primarily CNS-specific, the mapping of Panx2 protein distribution in other tissues has not been undertaken.

In this study, we compared Panx2 gene transcription and protein expression profiles in mouse tissues using a combination of real-time qPCR, Western blot and immunofluorescence. Our results reveal that Panx2 mRNA and protein levels are not correlated and demonstrate that Panx2 protein expression is more ubiquitous than initially predicted.

## Materials and methods

### Animal care

All experiments were performed in accordance with the guidelines established by the Canadian Council on Animal Care and were approved by the University of British Columbia Animal Care Committee (protocol number A11-0169).

### Antibodies

The two Panx2 mouse monoclonal antibodies (clones N121A/1 and N121A/31) were generated by UC Davis/NIH NeuroMab Facility (Davis, CA, USA) using an immunogen made of the entire rat Panx2 protein sequence (accession number P60571) minus the first 10 amino acids. Both clones were used at 20 µg/mL for immunofluorescence and 5 µg/mL for Western immunoblotting or dot blotting. The rabbit anti-Panx1 polyclonal antibody was generously provided by Dr. Dale Laird from the University of Western Ontario (London, ON, Canada) and was used at 2 µg/mL for immunofluorescence and 0.4 µg/mL for Western immunoblotting. The rabbit anti-GFAP (Sigma, St. Louis, MO, USA) was used at 1:500. Purified immunoglobulin from non-immunized mouse was obtained from Jackson Immunoresearch (cat# 015-000-003; West Grove, PA, USA) and was used at the same concentration as the anti-Panx2 antibodies. AlexaFluor- and HRPO-conjugated goat secondary antibodies were obtained from Invitrogen (Carlsbad, CA, USA) and Sigma (St. Louis, MO, USA) respectively.

### Cell culture

Wild-type C6 glioma cells as well as C6-Panx1GFP, C6-Panx2 and C6-Panx2GFP stable transfectants were cultured as previously described (Lai et al., 2007, 2009). Briefly, cells were grown in low glucose DMEM (Sigma-Aldrich, St. Louis, MO, USA) containing 10% fetal bovine serum, 10 units/mL penicillin, and 10 µg/mL streptomycin at 37°C and 5% CO_2_. Primary cultures of astrocytes were prepared as previously described (Le et al., 2014). Briefly, cortices were dissected from early postnatal (P0–P1) mouse pups, freed of meninges, minced and mechanically triturated in DMEM. The cell suspension was then strained through a 70 µm filter and seeded into T75 flasks (2 cortices/flask). Cells were cultured in DMEM (Sigma-Aldrich, St. Louis, MO, USA) containing 10% fetal bovine serum, 10 units/mL penicillin, and 10 µg/mL streptomycin at 37°C and 5% CO_2_ and the medium was initially replaced 3 days after plating and every other day subsequently. After 7–8 days, the flasks were vigorously shaken to remove loosely attached cells and primary astrocytes were harvested with trypsin-EDTA (Invitrogen, Carlsbad, CA, USA) and frozen in DMEM, 10% FBS, and 8% DMSO. Frozen astrocytes were thawed and plated on glass coverslips coated with poly-L-ornithine (0.01% solution, Sigma-Aldrich, St. Louis, MO, USA). Cultures were maintained for 5, 10 or 15 days prior to staining. The percentage of Panx2-positive astrocytes was defined as the number of cells that stained positively for Panx2 divided by the number of nuclei. For each time point, that ratio was calculated by averaging the values obtained from three coverslips with 10 field of views (168 × 225 µm) per coverslip.

### Epitope mapping

A library of 70 overlapping peptides covering the entire sequence of the rat Panx2 (Uniprot accession number P60571) minus the four transmembrane domains was obtained from Genscript (Piscataway, NJ, USA). Peptides were 15 amino acids in length with a 7 amino acids overlap. A total of 100 µg of peptide was pre-incubated overnight at 4°C in 100 µL of dot-blot buffer (8 M Urea, 100 mM NaH_2_PO_4_, 10 mM Tris, pH 8.0) containing 10 µg bovine serum albumin. Peptides were then dot-blotted on nitrocellulose membrane (Bio-Rad, Hercules, CA, USA), washed with sodium phosphate buffered saline (PBS, pH 7.4), dried at 37°C and blocked for 1 h in milk solution (4% nonfat milk, 20 mM Tris, 150 mM NaCl pH 7.4). The membrane was then immunoprobed for 2 h at room temperature with the primary antibody followed by HRPO-conjugated secondary antibodies (Sigma, St. Louis, MO, USA) for 1 h at room temperature.

### RNA isolation and real-time quantitative PCR

Three 3–5 month old mice were deeply anesthetized by intraperitoneal injection of sodium pentobarbital and perfused transcardially with 10–15 mL of PBS (pH 7.4) followed by 10–15 ml of aqueous ammonium sulfate solution (5.3 M ammonium sulfate, 25 mM sodium citrate, 10 mM EDTA, pH5.2) to precipitate degenerative RNases. Organs were rapidly harvested and stored at −80°C in the same solution. Total RNA was harvested from 50 mg of tissue using Trizol Reagent (Invitrogen, Carlsbad, CA, USA) according to the manufacturer’s directions. Air-dried RNA samples were re-solubilized in DEPC-treated double distilled H_2_O and RNA quantity and purity was assessed using a NanoDrop 1000 Spectrophotometer (Thermo Scientific, Waltham, MA, USA). All samples had A260/280 and A260/230 ratios above 1.9 and 2.3 respectively. A total of 500 ng per sample was reverse transcribed into cDNA in a 10 µL reaction volume using qScript (Quanta Biosciences, Gaithersburg, MD, USA) according to the manufacturer’s instructions. Real-time qPCR was performed in 18 µL reaction volume containing 45 ng of cDNA and 0.4 µM primers diluted in 2× Fast Plus EvaGreen^®^ qPCR Master Mix (Biotium Inc., Hayward, CA, USA). The following primer pairs were used to amplify Panx2 cDNA (forward 5′-AGAAGGCCAAGACTGAGGCG-3′ and reverse 5′- GGAGCATCTTTGGTGGGTGC-3′) and the reference gene DNA-directed RNA polymerase II subunit (RPB1) (Polr2a) cDNA (forward 5′- AGCTGGTCCTTCGAATCCGC-3′ and reverse 5′- TGGACTCAATGCATCGCAGGA-3′). Primers were designed to span an exon junction to prevent amplification of genomic DNA. Samples were amplified in duplicate using the CFX96 Real-Time PCR Detection System (Bio-Rad, Hercules, CA, USA) with the following cycling scheme: 2 min at 95°C followed by 50 cycles consisting of 5 s denaturation at 95°C, 5 s annealing at 60°C and 25 s elongation at 72°C. Raw data were exported as text files and analyzed with the qPCR package for R (Ritz and Spiess, 2008). Amplification efficiencies and Cy0 crossing points (Guescini et al., 2008) were calculated from a 5-parameter log-logistic model fitted to the raw fluorescence data to accommodate asymmetrical amplification curves (Spiess et al., 2008). Values from duplicated qPCR runs were averaged. Expression ratios were calculated from three biological replicates by the Pfaffl method to correct for variation in PCR efficiency (Pfaffl, 2001) and normalized against the reference gene Polr2a. Spinal cord mRNA levels were used as baseline to compare Panx2 expression across tissues. Propagation of error was estimated by a Monte Carlo simulation with 10,000 iterations as described in the qPCR package documentation.

### Protein isolation and western blotting

Organs were quickly collected after transcardial perfusion with PBS, flash frozen in liquid nitrogen and stored at −80°C until needed. Tissues or cells were homogenized in RIPA buffer (150 mM NaCl, 50 mM Tris-HCl pH 8.0, 0.5% Sarkosyl, 1% IGEPAL, 0.1% SDS) containing protease inhibitors (Pierce, Rockford, IL, USA) and phosphatase inhibitors (Sigma, St. Louis, MO, USA). Protein concentration was determined using a bicinchoninic acid (BCA) assay kit (Pierce, Rockford, IL, USA) and 50 µg was separated on 10% Tris-glycine SDS-PAGE gels containing 0.5% 2,2,2-trichloroethanol (TCE; Sigma, St. Louis, MO, USA). Upon electrophoresis completion, protein bands were visualized at 300 nm on an AlphaImager 3400 transilluminator (AlphaInnotech, San Leandro, CA, USA) as previously described (Ladner et al., 2004) and electroblotted on nitrocellulose (NCL) membrane (Bio-Rad, Hercules, CA, USA). Protein bands on NCL were re-visualized under UV for quantification and total protein normalization (Gürtler et al., 2013). For analysis which did not require quantification, TCE was omitted and PVDF membranes were used (Bio-Rad, Hercules, CA, USA). Membranes were blocked at room temperature for 1 h in milk solution (4% nonfat milk, 20 mM Tris, 150 mM NaCl, pH 7.4) and probed with primary antibodies at 4°C overnight followed by HRPO-conjugated secondary antibodies (Sigma, St. Louis, MO, USA) for 1 h at room temperature. All antibodies were diluted in blocking solution. HRPO activity was visualized with Amersham ECL Prime Western Blotting Detection Reagent (GE Healthcare Life Sciences, Pittsburgh, PA, USA) or SuperSignal West Femto Chemiluminescent Substrate (Thermo Scientific, Waltham, MA, USA) and exposed on Bioflex Econo films (Clonex, Markham, ON, Canada). Image acquisition for Western blot quantification was done as previously described (Gassmann et al., 2009). Briefly, film images were acquired on an AlphaImager 3400 (AlphaInnotech, San Leandro, CA, USA) under stable transillumination and fitted with CCD camera lacking automatic gain control. Final 16-bit 1392×1040 pixel images were corrected for shading to compensate for non-homogenous illumination and densitometry analysis was performed using the Image Studio Lite software (LI-COR, Lincoln, NE, USA). Panx2 protein ratios were calculated by dividing the band density of each tissue by the band density of the spinal cord. A tissue lysate from spinal cord was resolved on each gel to permit between gel comparisons. Panx2 protein ratios were calculated from three independent biological replicates.

### Immunofluorescence

Three 3–5 month old mice were transcardially perfused with PBS followed by 10–15 mL 10% formalin (Fisher) or 4% paraformaldehyde (PFA) in PBS. Tissues were rapidly harvested and postfixed overnight at 4°C in the same fixative. Tissues were equilibrated at 4°C in 30% sucrose in PBS containing 0.05% sodium azide for cryoprotection, embedded in Tissue-Tek O.C.T. compound (Sakura Finetek, Torrance, CA, USA), frozen, cryosectioned at 10 µm thickness and air-dried. Tissue sections were rehydrated in 0.1 M phosphate buffer (PB, pH 7.4), post-fixed with 4% PFA for 10 min and washed twice with PB. Antigen retrieval was performed by incubating the sections in 10 mM sodium citrate (pH 8.5) at 80°C for 30 min. Sections were cooled to room temperature, washed with PB and treated with 1% sodium borohydride in PB for 30 min. After several washes in PB, samples were blocked for 1 h at room temperature with 5% goat serum in PB containing 0.2% Triton X-100 and incubated overnight at 4°C in primary antibody diluted in 1% goat serum in PB. Sections were then washed in PB and incubated for 1 h at room temperature with the appropriate AlexaFluor 488 or 568 secondary antibodies (Invitrogen, Carlsbad, CA, USA), followed by mounting in ProLong Gold antifade reagent with DAPI (Invitrogen). Cells grown on coverslips were simply fixed with 4% PFA for 15–20 min prior blocking and immunostaining. Imaging was performed on a Leica TCS SP5 confocal microscope (Leica, Mannheim, Germany). When indicated, images were further processed with an iterative Lucy-Richardson deconvolution algorithm (Vonesch and Unser, 2008). All images were displayed as individual optical sections taken from a z-stack and not as maximal projection. All image acquisition and post-acquisition processing steps were kept constant when comparing sections stained with anti-Panx2 antibodies and immunoglobulins from non-immunized animal.

## Results

### Characterization of two novel monoclonal antibodies specific for Panx2

**Figure 1:**
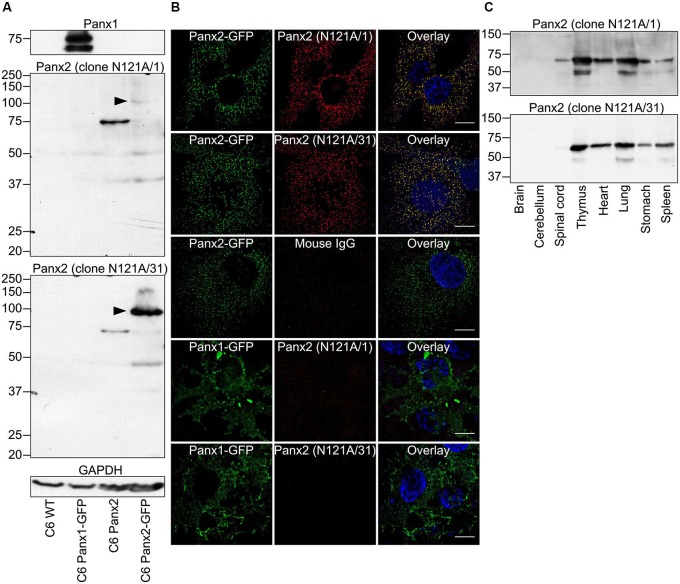
**Specificity of two novel anti-Panx2 monoclonal antibodies**. Two anti-Panx2 monoclonal antibodies (clones N121A/1 and N121A/31) showed high specificity both in Western blot **(A,C)** and immunolabeling **(B)**. **(A)** Both clones detected a single band of the proper size in C6 cells overexpressing Panx2 but did not cross-react with Panx1 or identify unspecific protein bands in wild-type C6 cells. The molecular weight of the protein identified by both clones shifted by ~27 kDa when the Panx2 C-terminal was tagged with GFP (black arrowheads). Interestingly, the avidity of clone N121A/1 was drastically reduced when the Panx2 was tagged with GFP. This is explained by the fact that clone N121A/1 recognizes an epitope immediately adjacent to the GFP tag (Figure 2). **(B)** Immunolabeling signal from both clones co-localized with GFP fluorescence emitted from Panx2GFP but not Panx1GFP when expressed in C6 cells. Immunoglobulin from non-immunized mouse did not give any signal thereby ruling out the possibility of unspecific binding of mouse IgG. Scale bars = 20 µm. **(C)** Both clones were equally specific in Western blot performed with various tissue lysates and identified a protein of ~70 kDa. The occasional detection of a ~50 kDa band in some tissues suggest that the antibodies might recognize Panx2 degradation products or alternatively that the anti-mouse secondary antibody detects endogenous immunoglobulin heavy chains.

**Figure 2:**
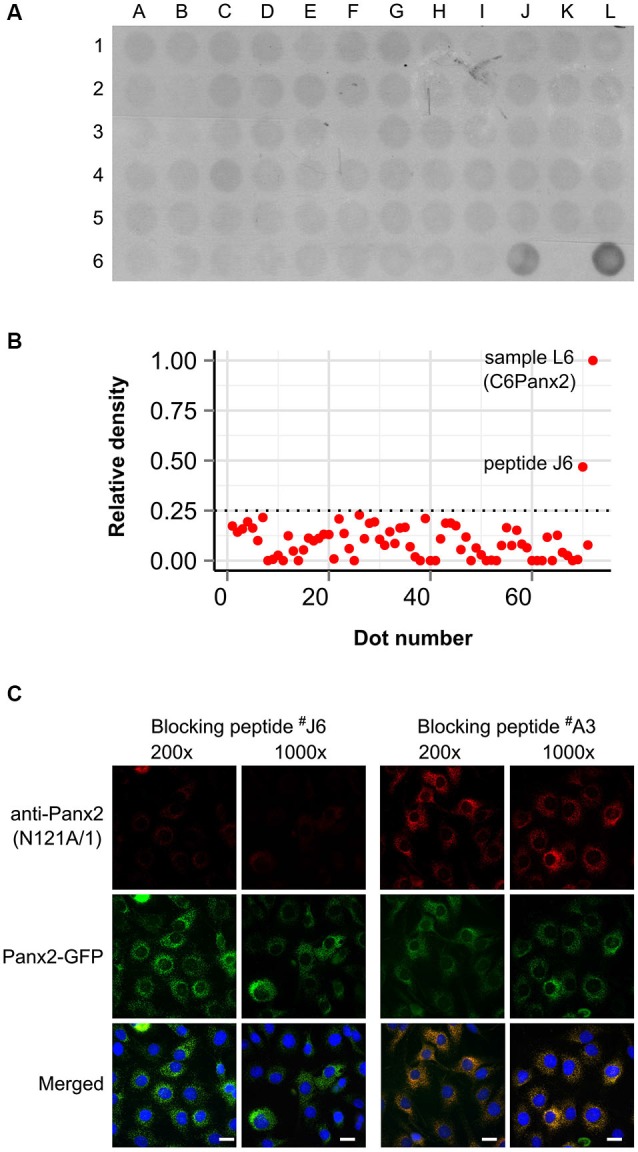
**Clone N121A/1 recognizes an epitope located within the last 15 amino acids of the Panx2 C-terminal. (A)** A library of 70 overlapping peptides spanning the entire Panx2 amino acid sequence minus the four transmembrane domains was dot blotted (A1 to J6) and immunoprobed with the N121A/1 anti-Panx2 antibody. A total of 25 µg of protein lysate from wild-type C6 and C6Panx2 glioma cells (K6 and L6 respectively) was also dot blotted alongside peptides to provide negative and positive control respectively. The clone N121A/1 specifically recognized C6Panx2 protein lysate and peptide J6 corresponding to the last 15 amino acids of the Panx2 C-terminal (TFEEPRTVVSTVEF). **(B)** Densitometry analysis of the dot blot staining shown in **(A)**. Only peptide J6 showed signal above threshold. Values were normalized against the density of the C6Panx2 sample. **(C)** The epitope identity was further confirmed by a blocking peptide assay. Pre-absorbing the N121A/1 anti-Panx2 antibody with a 200- or 1000-fold molar excess of peptide J6 dramatically reduced immunolabeling while pre-absorption with a peptide randomly selected along the Panx2 amino acid sequence (peptide A3) did not alter the labeling intensity. Scale bars = 20 µm.

We initially characterized the selectivity of two novel anti-Panx2 monoclonal antibodies (N121A/1 and N121A/31) by showing that both clones identified a single band of the proper size in C6 cells overexpressing rat Panx2 (Figure 1A). Importantly, none of the antibody cross-reacted with Panx1 (Figure 1A) which has been shown to be co-expressed with Panx2 in the CNS (Vogt et al., 2005). Selectivity was further supported by an electrophoretic mobility assay showing that the band identified by both clones shifted by ~27 kDa when the Panx2 C-terminal tail was tagged with GFP (Figure 1A). Intriguingly, tagging Panx2 with GFP decreased the avidity of clone N121A/1. Using a library of overlapping peptides covering the amino acid sequence of Panx2, we mapped the epitope of clone N121A/1 to the last 15 amino acids of the Panx2 C-terminal tail (Figure 2). It is therefore likely that the addition of a GFP tag adjacent to the epitope caused steric hindrance and reduced the avidity of clone N121A/1. We also tested the antibodies by immunofluorescence and showed that the labelling from both clones overlapped with the GFP fluorescence signal emitted by Panx2-GFP but not by Panx1-GFP (Figure 1B).

To test whether the novel antibodies could also be used on samples expressing endogenous Panx2 protein we immunoprobed protein lysates prepared from eight tissues and separated by SDS-PAGE (Figure 1C). Our results show that both clones recognized a band of approximately 70 kDa corresponding to the expected size of endogenous Panx2 as previously reported (Zappalà et al., 2007; Figure 1C). Overall our results identified two different commercial monoclonal antibodies specific for Panx2. Both clones recognize rat and mouse Panx2 and we also successfully used clone N121A/1 to detect human Panx2 (data not shown). To avoid redundancy and because both clones were equally specific we selected clone N121A/1 for subsequent analysis.

### Panx2 has a ubiquitous protein expression profile

**Figure 3:**
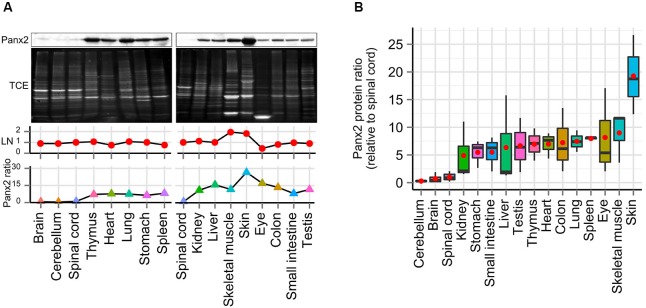
**Panx2 protein is ubiquitously expressed. (A)** Panx2 protein levels were semi-quantified in 16 tissues using stain-free total protein quantification to normalize protein levels across samples. Following exposure to TCE and UV, protein bands electroblotted on nitrocellulose (NTC) were visualized by fluorescence (TCE, second panel) and loading normalization (LN) was performed by dividing the fluorescence density from an entire individual lane by the total fluorescent density measured from the spinal cord lane on the corresponding NTC membrane (LN, third panel). Normalized Panx2 protein ratios were expressed relative to Panx2 levels found in the spinal cord (fourth panel). **(B)** Panx2 protein ratios were calculated from three different mice (relative to spinal cord, red dots represent mean values). Panx2 protein levels were lower in the CNS than any other tissues.

Interestingly our initial results showed substantial Panx2 protein amount outside the nervous system (Figure 1C). To further compare the Panx2 protein expression profile of different tissues, we carried out semi-quantitative densitometry analysis on protein lysates obtained from 16 tissues, separated by SDS-PAGE and immunoprobed for Panx2 (Figure 3A). Because the expression of common reference proteins was subjected to important fluctuations across tissues (data not shown), a stain-free total protein normalization strategy was employed to control for even loading as previously described (Ladner et al., 2004; Gürtler et al., 2013). Briefly, we incorporated TCE in the gel formulation which, upon ultraviolet (UV) irradiation, catalyzes a covalent reaction with tryptophan residues. This reaction emits fluorescence that can be imaged and documented in gel and following protein transfer on membranes (Ladner et al., 2004; Gürtler et al., 2013). The Panx2 staining density for each tissue was then normalized against the intensity of the TCE-fluorescence measured for the entire lane after protein transfer on nitrocellulose membrane (Figure 3A). Our data indicate that Panx2 protein is present at substantial levels in every tissue studied (Figures 3A,B). More surprisingly, in contradiction with current predictions, Panx2 protein was lower in the nervous system than in any other tissues (Figure 3B).

### Transcriptional activity does not predict Panx2 protein levels

**Figure 4:**
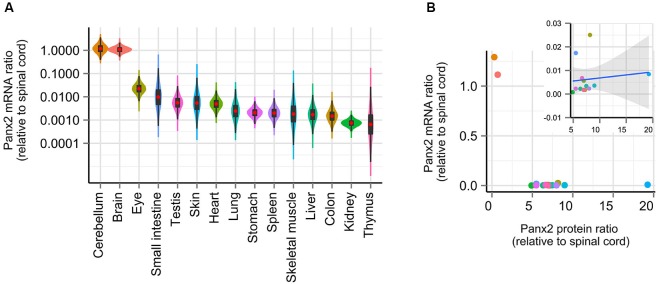
**Panx2 transcriptional activity does not correlate with protein levels. (A)** Panx2 mRNA levels were measured by real-time qPCR in 16 tissues and expressed relative to Panx2 mRNA levels found in the spinal cord. Error propagation was estimated by a Monte Carlo simulation and data distribution represented using a combination of violin and box plots (red dots represent mean values). Data were plotted on a logarithmic scale. Panx2 mRNA levels were several orders of magnitude higher in the CNS than in any other tissues. **(B)** Panx2 protein and mRNA levels were not correlated. No significant correlation was observed even after values from the CNS were eliminated (inset).

Gene profiling studies have reported that Panx2 mRNA expression is largely restricted to the CNS in human (Baranova et al., 2004), rat and mouse (Bruzzone et al., 2003; Dvoriantchikova et al., 2006) and zebrafish (Zoidl et al., 2008; Bond et al., 2012) but a similar profiling study has not been completed in mouse. To determine whether our observations on Panx2 protein expression could be explained by species-specific Panx2 transcriptional activity, we compared the Panx2 transcription profile using RNA isolated from 16 mouse tissues, reverse-transcribed into cDNA and analyzed by real-time qPCR. Although primers were designed to span an exon junction, end-point PCR was initially performed using non-transcribed RNA as template to confirm the absence of genomic DNA amplification (data not shown). We also tested the specificity of our primer pair by visualizing the amplification product by gel electrophoresis and by analyzing the amplicon’s melting curve (data not shown). Our results are in accordance with previous studies (Bruzzone et al., 2003; Baranova et al., 2004; Dvoriantchikova et al., 2006; Zoidl et al., 2008; Bond et al., 2012) and showed that Panx2 transcriptional activity largely predominates in the CNS (Figure 4A). Panx2 transcript levels detected in non-neural tissues were several orders of magnitude lower than in the CNS (Figure 4A). Furthermore, we demonstrated that there is no significant correlation between Panx2 transcript and corresponding protein levels (Figure 4B).

Our results showed that Panx2 mRNA and protein levels are not correlated when compared across different tissues but does not exclude a possible correlation within a specific tissue as opposed to between different tissues. This scenario appears unlikely however as we have shown that Panx2 protein levels remain surprisingly constant in the brain over a developmental period during which Panx2 mRNA levels have been shown to be temporally up-regulated (data not shown) (Vogt et al., 2005). Overall, these results suggest that regulatory mechanisms unrelated to transcriptional activity must also control Panx2 protein levels and indicate that Panx2 protein levels cannot be directly inferred from the quantification of its transcript levels.

### Panx2 protein is localized to cytoplasmic compartments

**Figure 5:**
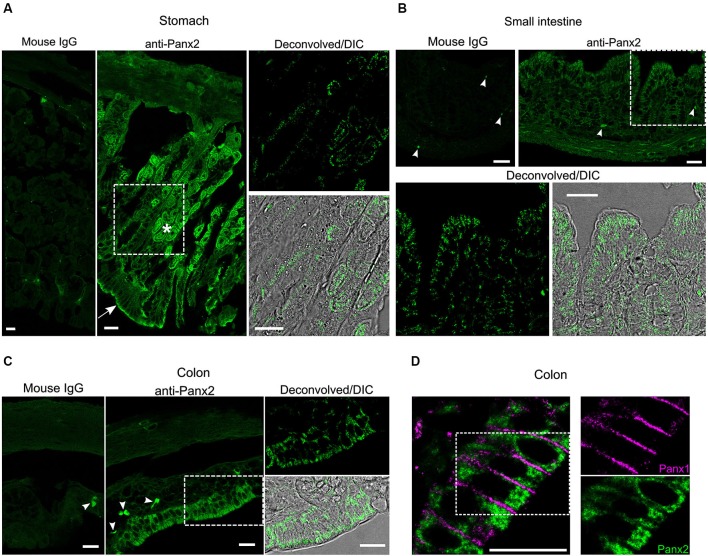
**Panx2 protein is expressed in the gastrointestinal tract**. Sections of mouse stomach **(A)** small intestine **(B)** and colon **(C)** were stained with an immunoglobulin from non-immunized mouse (Mouse IgG panels) or the N121A/1 monoclonal anti-Panx2 antibody (anti-Panx2 and Deconvolved/DIC panels). Panx2 protein was heavily expressed in the parietal (asterisk) and epithelial (arrow) cells of the stomach **(A)** and the epithelial cells of the small **(B)** and large **(C)** intestine. Panx2 distribution was primarily perinuclear and cytoplasmic. No specific staining was observed with the IgG from non-immunized mouse. Putative lymphocytes expressing endogenous immunoglobulins were occasionally labeled by the anti-mouse secondary antibody (arrowheads). **(D)** Panx1 and Panx2 showed distinct subcellular distribution in the colon. Scale bars: 20 µm.

We next characterized the expression and distribution of Panx2 in different tissues by immunofluorescence. In the gastrointestinal tract, an important population of glandular and epithelial cells displayed strong Panx2 immunoreactivity (Figure 5). Parietal cells, which secrete gastric acid, and the apical surface of epithelial cells of the stomach were strongly reactive for Panx2 (Figure 5A). In the small and large intestine, a population of columnar epithelial cells were also strongly reactive for Panx2 (Figures 5B,C). As Panx1 has also been shown to be expressed in the columnar epithelial cells of the human colon (Diezmos et al., 2013), we tested whether Panx1 and Panx2 could co-localize in these cell types. Interestingly, Panx1 and Panx2 did not co-localize but showed quite different subcellular distribution patterns (Figure 5D). Panx1 expression was largely restricted to the plasma membrane between the epithelial cells. In contrast, Panx2 was not discernible at the plasma membrane but remained largely confined to the cytoplasmic area (Figure 5D).

**Figure 6:**
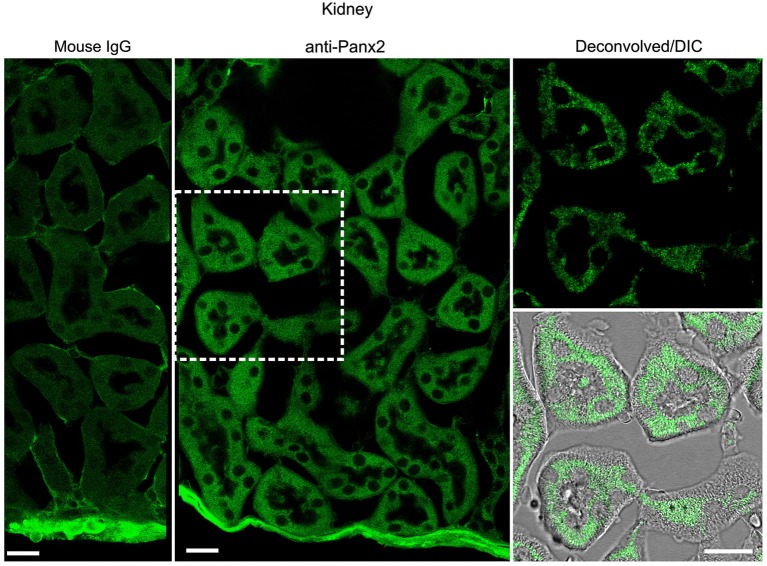
**Cuboidal cell from kidney tubule express Panx2 protein**. Sections of mouse kidney labeled with an immunoglobulin from non-immunized mouse (Mouse IgG left panel) or the N121A/1 monoclonal anti-Panx2 antibody (middle and right panels). Panx2 protein was distributed in the cytoplasm of the epithelial cells lining the lumen of renal tubule but was not discernible at the plasma membrane. Scale bars: 20 µm.

**Figure 7:**
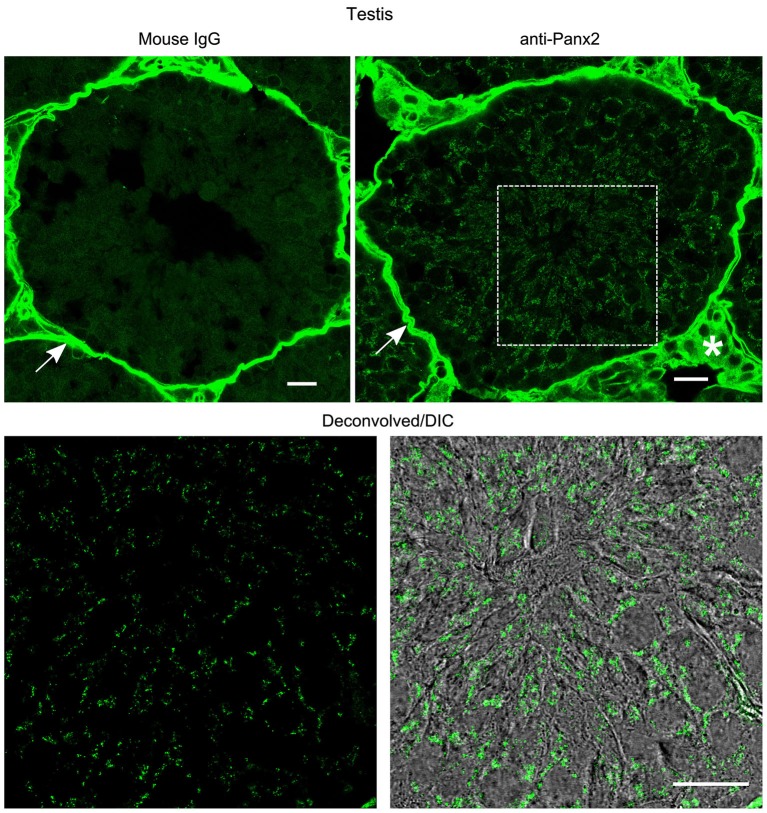
**Germ cells from mouse testis express Panx2 protein**. Sections of mouse testis were labeled with an immunoglobulin from non-immunized mouse (Mouse IgG panel) or the N121A/1 monoclonal anti-Panx2 antibody (anti-Panx2 and Deconvolved/DIC panels). Panx2 protein was localized in the cytoplasm of germ cells in the seminiferous epithelium. Smooth muscle cells (arrow) and interstitial tissue (asterisk) showed high autofluorescence levels. Scale bars: 20 µm.

In the kidney, cuboidal cells forming the single layered epithelium of tubules were strongly labeled (Figure 6) whereas glomeruli cells were not (data not shown). Panx2 staining was predominantly cytoplasmic and could not be detected at the plasma membrane. Similarly, germ cells from testis seminiferous tubules showed abundant perinuclear and cytoplasmic but no plasma membrane staining (Figure 7).

**Figure 8:**
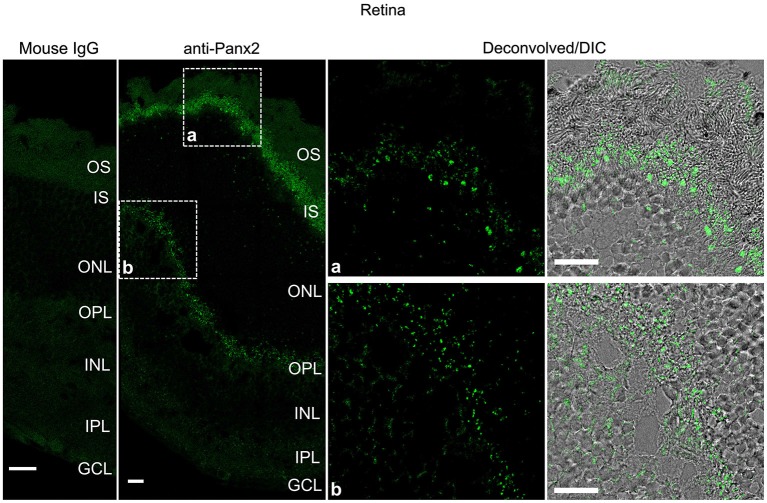
**Panx2 protein is expressed in the photoreceptor inner segment and outer plexiform layer of the mouse retina**. Sections from mouse retina were labeled with an immunoglobulin from non-immunized mouse (Mouse IgG panel) or the N121A/1 monoclonal anti-Panx2 antibody (anti-Panx2 and Deconvolved/DIC panels). Panx2 protein was primarily expressed in the photoreceptor inner segment (IS) (inset a) or the outer plexiform layer (OPL) (inset b). Panx2 clustered in small to large aggregates which appeared to be primarily cytoplasmic. However, because of the high degree of cellular compaction we could not reach definitive conclusions regarding Panx2 subcellular distribution in the retina. OS: photoreceptor outer segment, IS: photoreceptor inner segment, ONL: outer nuclear layer, OPL: outer plexiform layer, INL: inner nuclear layer, IPL: inner plexiform layer, GCL: ganglion cell layer. Scale bars: 20 µm.

Panx2 immunoreactivity displayed a distinct pattern in the mouse retina (Figure 8). Photoreceptor inner segments protruding into the subretinal space were densely decorated with Panx2-labeled aggregates (Figure 8). Only sparse immunoreactivity was observed in the outer nuclear layer which forms the compact layer containing photoreceptor cell bodies (Figure 8). Substantial staining was also observed in the outer plexiform layer which comprises a dense network of neuronal synapses formed between photoreceptors and bipolar and horizontal cell dendrites (Figure 8).

**Figure 9:**
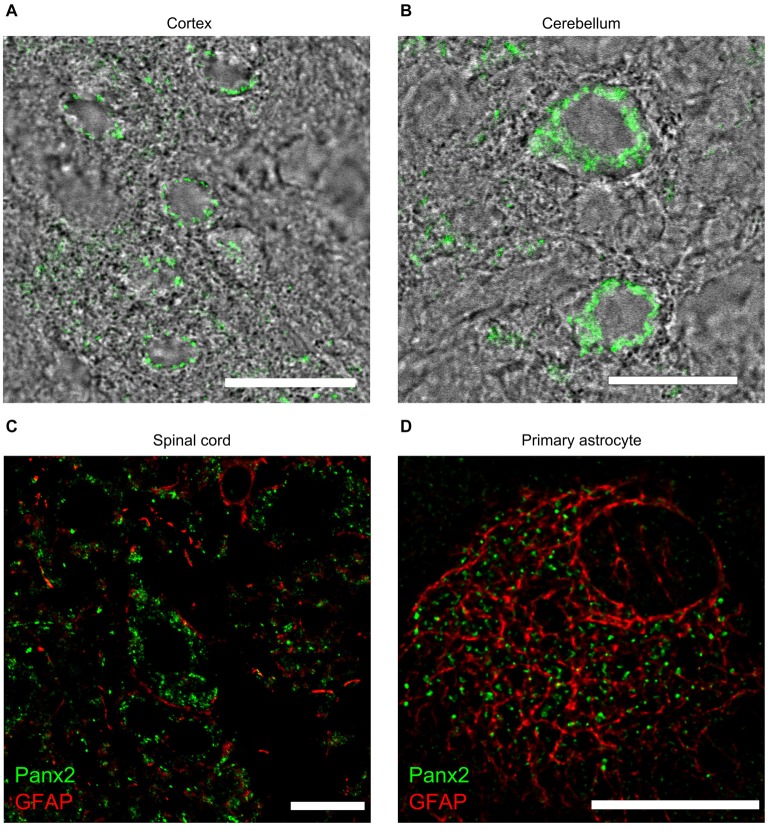
**Panx2 protein localizes in the cytoplasm of CNS neurons**. Panx2 heavily labeled the cytoplasm, but not the plasma membrane, of cortical neurons **(A)** Purkinje cells **(B)** and spinal cord motoneurons **(C)**. Panx2 was not detected in GFAP-positive astrocytes in the brain (data not shown) or the spinal cord **(C)** but was detected in over 60% of primary astrocytes cultured for 5 days **(D)**. Scale bars: 20 µm.

Despite showing lower Panx2 protein levels than any other tissues (Figure 3), Panx2 immunoreactivity was easily distinguishable by immunofluorescence in the CNS (Figure 9) and showed a complex expression pattern as previously reported (Zappalà et al., 2007). Panx2 was widely distributed in the cytoplasm of neurons throughout the CNS but was not readily detected in astrocytes *in vivo* (Figures 9A–C). Interestingly, we showed that the majority of primary astrocytes (63.8 ± 0.9%) expressed cytoplasmic Panx2 at 5 days *in vitro* (Figure 9D). However, the percentage of Panx2-positive astrocytes rapidly declined after 10 and 15 days *in vitro* (6.9 ± 1.2% and 7.3 ± 1.1%) hereby suggesting that Panx2 is expressed by immature but not mature astrocytes. That observation could explain the up-regulation of Panx2 expression seen in astrocytes following ischemia (Zappalà et al., 2007) as ischemia is characterized by astrocyte proliferation.

## Discussion

This study reveals that Panx2 protein is more ubiquitous than initially predicted. By performing real-time qPCR and semi-quantitative Western blot analysis on a panel of mouse tissues, we showed that fluctuations in Panx2 mRNA abundance do not predict changes in Panx2 protein levels. We showed that Panx2 protein levels are surprisingly more abundant in non-neural tissues than in the CNS; an observation opposite to Panx2 transcriptional activity which is weak in non-neural tissues and largely predominant in the CNS. The ubiquitous expression of Panx2 protein suggests a more fundamental function than the CNS-specific role which was originally proposed. Although the exact function of Panx2 remains elusive, based on our *in vivo* immunofluorescence results we hypothesize that Panx2 channels do not significantly contribute to communication exchange between the intracellular and extracellular spaces but rather control intracellular signaling through cytoplasmic compartments.

### Absence of correlation between Panx2 mRNA and protein levels

The initial Panx2 gene expression profiles were obtained from Northern blots using commercial rat (Bruzzone et al., 2003) and human (Baranova et al., 2004) mRNA. Although notable differences exist between the two studies, both groups reported that Panx2 mRNA is largely predominant in the CNS; an observation that was subsequently confirmed in zebrafish using real-time qPCR (Zoidl et al., 2008; Bond et al., 2012). Our results show that Panx2 mRNA follows a similar expression profile in the mouse since Panx2 transcript levels are 40 to over 1600 times higher in the CNS than in other tissues. Because of this dramatic disparity in Panx2 mRNA expression it has long been assumed that Panx2 protein was preferentially, if not exclusively, expressed in the CNS.

However, in almost every organism steady-state transcript concentrations only partially correlate with protein expression levels (de Sousa Abreu et al., 2009) and the assumption that transcripts can predict protein abundances has been heavily challenged. Post-transcriptional regulatory mechanisms have overwhelming influence on changes observed at the proteome level (Foss et al., 2011) and protein levels cannot be accurately extrapolated from transcript levels because several factors unrelated to transcriptional control also directly influence protein levels. For example, protein degradation rate has been shown to influence the correlation between transcripts and corresponding protein levels as stable proteins are less affected by perturbations in mRNA levels than proteins with high turnover rates (Raj et al., 2006). Hence, the long half-life of Panx proteins (Penuela et al., 2007) could efficiently buffer important fluctuations in mRNA levels and decrease the impact of Panx2 transcripts on Panx2 protein levels. Bearing this information in mind, it is safe to affirm that variations of Panx2 transcript levels should be interpreted restrictively, without assuming equivalent changes at the protein level.

Mass-spectrometry-based proteomics can perform large-scale unbiased analyses of biological systems and examine which genes are translated into proteins in specific tissues. Recently, two groups assembled and published mass-spectrometry-based drafts of the human proteome into databases available online for real-time analysis (Kim et al., 2014; Wilhelm et al., 2014). Interestingly, unique Panx2 peptides were identified in the ileum, colon and ovary (Wilhelm et al., 2014) as well as the gut, spinal cord, urinary bladder, liver, ovary, testis and prostate (Kim et al., 2014). Although substantial improvements are still needed to achieve a complete and quantitative proteome coverage, these independent studies nonetheless corroborate our results and demonstrate that Panx2 protein expression is not restricted to the CNS.

It is important to note that Panx2 protein ratios showed high variability in some tissues (Figure 3B). This is more likely attributable to the limited dynamic range of the chemiluminescence technique that was used for the quantification of Panx2 protein expression. An alternative would have been to use a ratiometric analysis based on infrared detection of protein bands to increase the linear detection range and increased reproducibility (Zellner et al., 2008).

### Panx2: a cytoplasmic unusual suspect

Technical reasons such as prevalent autofluorescence prevented the analysis of certain tissues by immunofluorescence. Nonetheless, our study shows that Panx2 protein was heavily distributed in the cytoplasmic compartment and could not be readily detected at the plasma membrane in all tissues analyzed by immunofluorescence (9 out of 16) or in cultured primary astrocytes expressing endogenous Panx2 protein. Previous studies had reported cytoplasmic Panx2 in transfected overexpression systems (Lai et al., 2009; Penuela et al., 2009; Bhalla-Gehi et al., 2010) or in neurons and neural progenitor cells (Zappalà et al., 2007; Swayne et al., 2010) but we are the first group to identify endogenous cytoplasmic Panx2 in such a large variety of tissues.

The unique intracellular distribution of Panx2 protein is in striking contrast with Panx1 and Panx3 proteins which are primarily localized at the plasma membrane (Penuela et al., 2007, 2009; Bhalla-Gehi et al., 2010). The cellular localization of Panx proteins is influenced by glycosylation (Penuela et al., 2009). All three Panx paralogs are glycosylated to a high mannose form in the endoplasmic reticulum (ER; Penuela et al., 2009; Bhalla-Gehi et al., 2010) but interestingly only Panx1 and Panx3 proteins form complex glycoprotein species requiring post-translational modifications occurring in the Golgi (Penuela et al., 2009; Bhalla-Gehi et al., 2010). Panx1 and Panx3 proteins follow a COPII-dependent ER to Golgi secretory pathway prior to being trafficked to the plasma membrane (Bhalla-Gehi et al., 2010). In contrast, the absence of complex glycosylated Panx2 suggests Panx2 protein follows a different trafficking pathway which might not involve transition through the Golgi and subsequent trafficking to the plasma membrane. Intriguingly, Panx2 has been shown to co-localize with the endolysosomal enriched mannose-6-phosphate receptor in N2a neuroblastoma cells expressing Panx2 tagged with GFP (Wicki-Stordeur et al., 2013). However, the study used transient overexpression of tagged Panx2 protein which might have resulted in the accumulation of misfolded or misassembled proteins and increased the likelihood of artifactual missorting in endomembrane compartments. Therefore, additional localization studies detecting endogenous Panx2 protein using a combination of different approaches are still needed for the accurate identification of Panx2-positive cytoplasmic compartments.

Others have detected putative Panx2 at the plasma membrane of mature primary hippocampal neurons (Swayne et al., 2010) or in cell types overexpressing Panx2 (Ambrosi et al., 2010). The ectopic expression of Panx1 and Panx2 in NRK cells has also been shown to increase Panx2 trafficking to the plasma membrane (Penuela et al., 2009). However, the physiological relevance of this increase in Panx2 at the plasma membrane is unclear because Panx1/Panx2 heteromeric channels are unstable (Ambrosi et al., 2010). Although we cannot exclude the possibility that undetectable levels of Panx2 are distributed at the plasma membrane in some cell types, we conclude that under physiological conditions Panx2 protein is primarily localized in the cytoplasmic compartment in most, if not all, tissues.

Gap junctions have traditionally been described as plasma membrane channels connecting the cytoplasm of adjacent cells or controlling the exchange of small molecules between the intracellular and extracellular spaces. Our results suggest that a different model must apply to Panx2 because its range of action seems to be restricted to the cytoplasmic milieu. Consequently, we hypothesize that Panx2 can modulate cell activity through non-conventional routes and novel intracellular signaling pathways. In that aspect, it is interesting to note that over-expression of Panx1 and Panx3 can form calcium permeable channels in the ER (Vanden Abeele et al., 2006; Ishikawa et al., 2011). As over-expression of Panx2 in C6 cells showed a prominent signal overlap with the ER (Lai et al., 2009) it is possible that Panx2 can also modulate ER calcium signaling. Moreover, although an essential property of gap junction proteins is their ability to oligomerize to form transmembrane channels, it should be emphasized that gap junctions also have channel-independent functions. For example, connexin 43 (Cx43) has recently been shown to control the biogenesis of autophagosomes through the sequestration of several autophagy-related proteins (Bejarano et al., 2014); a function independent of Cx43 channel activity. Since Panx2 has a long C-terminal tail (301 a.a) it is reasonable to suggest that protein-protein interactions involving its C-terminus are likely to play an important role in the function of Panx2. However, until the exact nature of Panx2 subcellular compartment remains unknown, formulating hypothesis regarding the function of Panx2 remains rather difficult.

Our understanding of Panx2 protein currently assumes that Panx2 function can be extrapolated from our knowledge of the other Panx proteins. More precisely, Panx2 is often perceived as a CNS-specific protein assuming a role complementary, if not redundant, to the function of Panx1 channel. However, our study shows that this assumption is misleading and unlikely to increase our knowledge on any of the Panx channels. Prior to our work, several studies investigating the role of Panx channels outside of the CNS focused exclusively on Panx1 and Panx3 but completely neglected the potential implication of Panx2. As our study shows that Panx2 protein expression is more ubiquitous than initially predicted it would be interesting to revisit these original studies while taking into account the presence of Panx2. This is especially important in the context of Panx1 knockout mice since the deletion of Panx1 could have compensatory effects by altering the expression level of Panx2. Another cautionary note needs to be highlighted regarding the techniques that are currently used to assay Panx2 functionality. Several studies use patch-clamping of the plasma membrane to address the functionality of Panx2 channels (for example see Bargiotas et al., 2011; Poon et al., 2014). Although we cannot totally exclude the presence of Panx2 at the plasma membrane, our study nonetheless shows that Panx2 protein is predominantly in cytoplasmic compartments. Consequently, Panx2 channel properties cannot be solely investigated through electrophysiological recordings at the plasma membrane.

## Conflict of interest statement

The authors declare that the research was conducted in the absence of any commercial or financial relationships that could be construed as a potential conflict of interest.

## Abbreviations

Cx: connexin
Inx: innexin
Panx: pannexin
qPCR: quantitative real-time PCR
GFAP: glial fibrillary acidic protein
HRPO: horseradish peroxidase
DEPC: diethylpyrocarbonate
Polr2a: DNA-directed RNA polymerase II subunit RPB1
SDS-PAGE: sodium dodecyl sulfate polyacrylamide gel electrophoresis
BCA: bicinchoninic acid
TCE: 2,2,2-trichloroethanol
NCL: nitrocellulose
PB: phosphate buffer.
